# Transient association between semen exposure and biomarkers of genital inflammation in South African women at risk of HIV infection

**DOI:** 10.1002/jia2.25766

**Published:** 2021-06-24

**Authors:** Janine Jewanraj, Sinaye Ngcapu, Farzana Osman, Veron Ramsuran, Maryam Fish, Andile Mtshali, Ravesh Singh, Leila E Mansoor, Salim S Abdool Karim, Quarraisha Abdool Karim, Jo‐Ann S Passmore, Lenine J P Liebenberg

**Affiliations:** ^1^ Centre for the AIDS Programme of Research in South Africa (CAPRISA) University of KwaZulu‐Natal Durban South Africa; ^2^ Department of Medical Microbiology School of Laboratory Medicine and Medical Sciences University of KwaZulu‐Natal Durban South Africa; ^3^ KwaZulu‐Natal Research Innovation and Sequencing Platform (KRISP) Durban South Africa; ^4^ Department of Microbiology National Health Laboratory Services KwaZulu‐Natal Academic Complex Inkosi Albert Luthuli Central Hospital Durban South Africa; ^5^ School of Nursing and Public Health University of KwaZulu‐Natal Durban South Africa; ^6^ Department of Epidemiology Columbia University New York NY USA; ^7^ Institute of Infectious Diseases and Molecular Medicine (IDM) University of Cape Town Cape Town South Africa; ^8^ National Health Laboratory Services Johannesburg South Africa

**Keywords:** semen, Y‐chromosome DNA, prostate‐specific antigen, female genital inflammation, cytokines, HIV

## Abstract

**Introduction:**

Semen induces mucosal changes in the female reproductive tract to improve pregnancy outcomes. Since semen‐induced alterations are likely short‐lived and genital inflammation is linked to HIV acquisition in women, we investigated the contribution of recent semen exposure on biomarkers of genital inflammation in women at high HIV risk and the persistence of these associations.

**Methods:**

We assessed stored genital specimens from 152 HIV‐negative KwaZulu‐Natal women who participated in the CAPRISA 008 trial between November 2012 and October 2014. During the two‐year study period, 651 vaginal specimens were collected biannually (mean five samples per woman). Cervicovaginal lavage (CVL) was screened for prostate‐specific antigen (PSA) by ELISA, whereas Y‐chromosome DNA (YcDNA) detection and quantification were conducted by RT‐PCR, representing semen exposure within 48 hours (PSA+YcDNA+) and semen exposure within three to fifteen days (PSA−YcDNA+). Soluble protein concentrations were measured in CVLs by multiplexed ELISA. T‐cell frequencies were assessed in cytobrushes by flow‐cytometry, and vulvovaginal swabs were used to detect common vaginal microbes by PCR. Linear mixed models adjusting for factors associated with genital inflammation and HIV risk were used to assess the impact of semen exposure on biomarkers of inflammation over multiple visits.

**Results:**

Here, 19% (125/651) of CVLs were PSA+YcDNA+, 14% (93/651) were PSA−YcDNA+ and 67% (433/651) were PSA−YcDNA−. Semen exposure was associated with how often women saw their partners, the frequency of vaginal sex in the past month, HSV‐2 antibody detection, current gonorrhoea infection and Nugent Score. Both PSA detection (PSA+YcDNA+) and higher cervicovaginal YcDNA concentrations predicted increases in several cytokines, barrier‐related proteins (MMP‐2, TIMP‐1 and TIMP‐4) and activated CD4+CCR5+HLA‐DR+ T cells (β = 0.050; CI 0.001 to 0.098; *p* = 0.046) and CD4+HLA‐DR+ T cells (β = 0.177; CI 0.016 to 0.339; *p* = 0.032) respectively. PSA detection was specifically associated with raised pro‐inflammatory cytokines (including IL‐6, TNF‐α, IP‐10 and RANTES), and with the detection of BVAB2 (OR = 1.755; CI 1.116 to 2.760; *p* = 0.015), *P*. *bivia* (OR = 1.886; CI 1.102 to 3.228; *p* = 0.021) and *Gardnerella vaginalis* (OR = 1.815; CI 1.093 to 3.015; *p* = 0.021).

**Conclusions:**

More recent semen exposure was associated with raised levels of inflammatory biomarkers and the detection of BV‐associated microbes, which declined by three to fifteen days of post‐exposure. Although transient, semen‐induced alterations may have implications for HIV susceptibility in women.

## 1 Introduction

Semen induces mucosal alterations at the female reproductive tract to improve pregnancy outcomes [1, 2, 3]. However, the contribution of semen‐associated changes to human immunodeficiency virus (HIV) risk in women remains unclear. Although an optimal vaginal environment has several defences to prevent infection, genital inflammation limits host defences [4, 5, 6, 7] and increases HIV infection risk, even by less fit viruses [4, 8]. Biomarkers of genital inflammation include elevated cervicovaginal cytokines, immune cell recruitment, alterations in barrier‐related proteins and increased microbial diversity [4, 5, 6, 7]. Semen contains several bioactive molecules and a diverse array of microbial communities [9, 10, 11, 12], which may alter HIV susceptibility in women by promoting genital inflammation. A better understanding of the female immune response during condomless sex and the semen properties that promote genital inflammation may aid in designing effective biomedical HIV prevention strategies in women.

Biomarkers that detect semen within vaginal specimens may be beneficial to characterize the effects of semen exposure on female genital inflammation and HIV risk. Prostate‐specific antigen (PSA) and Y‐chromosome deoxyribonucleic acid (YcDNA) detection are well‐characterized biomarkers of semen exposure [13, 14, 15, 16, 17, 18]. PSA is produced by the prostate gland, and detection in vaginal fluids at concentrations ≥1 ng/mL can be used to assess semen exposure at the female genital tract (FGT) even from vasectomized males [was used to detect PSA in 17, 18, 19]. In women, PSA is present for a short duration following condomless sex, and detection indicates semen exposure within 48 hours of cervicovaginal sampling [14, 18]. Alternatively, the measurement of YcDNA can be used as a more stable marker of semen exposure [15, 16]. YcDNA detection involves polymerase chain reaction (PCR) amplification of the testis‐specific protein Y‐encoded (TSPY) gene region and the sex‐determining region Y (SRY) gene region in the Y‐chromosome [15, 20]. YcDNA can be detected in vaginal fluid up to 15 days after condomless sex [15, 16, 21]. Furthermore, since YcDNA is detectable in the presence of spermatozoa, YcDNA quantities at the FGT may indicate sperm count and seminal protein concentrations.

Considering the transience of semen‐associated immune alterations in the FGT [1, 22], a pro‐inflammatory immune response to semen exposure may be better characterized using a biomarker of recent condomless sex. Here, we hypothesized that more recent semen exposure and higher cervicovaginal YcDNA concentrations would be associated with the inflammatory environment related to HIV risk in women. To test this hypothesis, we compared immune and microbial markers of genital inflammation among women with evidence of semen exposure within 48 hours (PSA+YcDNA+), three to fifteen days (PSA−YcDNA+) and no semen exposure within 15 days prior to genital sampling (PSA−YcDNA−). Additionally, we investigated the association between markers of genital inflammation and cervicovaginal concentrations of YcDNA, which may also reflect more recent sex and male protein concentrations at the FGT.

## 2 Methods

### 2.1 Study population and design

We enrolled 152 HIV‐negative women between 20 and 44 years of age from KwaZulu‐Natal who participated in the CAPRISA 008 study [23, 24, 25]. Women participated in the CAPRISA 008 study over two years, between November 2012 and October 2014, and were followed up for an average of 22 months [23, 25]. Demographic data were assessed at baseline, and vaginal specimens (including cervicovaginal lavage (CVL), cytobrushes and vulvovaginal swabs) were collected at enrolment and biannually at months 6, 12, 18, 24 and study exit (average 5 ± 1 visit; 651 genital specimens) as previously reported [24, 25, 26, 27]. Semen biomarkers were measured in vaginal specimens collected at each of the multiple study visits per participant. The detection of semen biomarkers indicated whether semen exposure occurred between zero to two and three to fifteen days before specimen collections at the respective visits. Participants provided informed consent for the specimen storage and use in future studies (BFC237/010). This study protocol was approved by the University of KwaZulu‐Natal Biomedical Research Ethics Committee (BE258/19).

### 2.2 Screening for semen in vaginal fluids

#### 2.2.1 PSA ELISA

The Human PSA‐total ELISA Kit (SIGMA‐ALDRICH™, St. Louis, MO, USA) was used to detect PSA in CVL supernatants according to the manufacturer’s protocol. Briefly, 100 µL of samples, standards and negative controls were examined in duplicate. The CVL specimens were considered PSA positive if detectable concentrations were above the lowest standard’s average concentration. Absorbance was measured at 450 nm with a VersaMax™ ELISA Microplate Reader. Detection of PSA within vaginal specimens indicated semen exposure within 48 hours before genital sampling.

#### 2.2.2 YcDNA detection and quantification

DNA extraction was conducted on CVL pellets using the MagNAPure LC DNA Isolation Kit I (Roche Applied Science, Indianapolis, IN, USA), as instructed by the manufacturer. The Human Y‐chromosome Quantification Kit (PrimerDesign Ltd, Chandler's Ford, UK) was used to detect a TSPY1 gene region present on the Y‐chromosome within the extracted DNA. The Y‐chromosome primer/probe mix and the *PrecisionFAST™* Mastermix were used according to the manufacturer's instructions (PrimerDesign Ltd). Amplification was conducted on the Applied Biosystems^®^ QuantStudio™ 5 real‐time (RT)‐PCR System (Thermo Fisher Scientific, Waltham, MA, USA). Detection of YcDNA within vaginal specimens indicated semen exposure within 15 days before genital sampling [15, 16]. The Quantifiler™ Trio DNA Quantification Kit from Applied Biosystems™ (Thermo Fisher Scientific) was used to quantify YcDNA concentrations in YcDNA+ CVL specimens as outlined in the manufacturer’s protocol. The assay simultaneously quantified the total amount of amplifiable human DNA and human male DNA in 10 µL of the sample.

#### 2.2.3 Investigation of soluble biomarkers of genital inflammation

The levels of genital cytokines and barrier‐related proteins were assessed in CVL supernatants using multiplexed ELISA assays. Cytokine concentrations were measured using the Bio‐Plex Pro™ Human Cytokine 21‐Plex and 27‐Plex kits (Bio‐Rad Laboratory, Hercules, CA, USA) as previously described (Table S1) [28]. Concentrations of matrix metalloproteinases (MMPs) and tissue inhibitors of metalloproteinases (TIMPs) were quantified using the MMP 9‐Plex, and TIMP 4‐Plex kits (Bio‐Rad Laboratory), respectively, as instructed by the manufacturer. MMP/TIMP measurements were performed on baseline samples (n = 136; Figure S1). All analyte concentrations were measured using the Bio‐Plex^®^ 200 system (Bio‐Rad Laboratory).

#### 2.2.4 Investigation of immune cell frequencies

Multiparametric flow cytometry was used to assess the expression of activation markers (CD38+ or HLA‐DR+), the maker of proliferation (Ki67+) and the HIV co‐receptor (CCR5+) on CD4+ T cells in cervical cytobrush‐derived specimens. The viability of cervical mononuclear cells (CMCs) was determined using the LIVE/DEAD™ Fixable Dead Cell Staining Kit (Invitrogen Life Technologies, Carlsbad, CA, USA) as outlined in the manufacturer's protocol. CMCs were treated with antibody‐conjugated fluorophores, washed and fixed (Table S2). Data were acquired on an LSRII flow cytometer (BD Immunocytometry Systems, San Jose, CA, USA) and analysed with FlowJo™ Software version 9.9 (Tree Star, Inc., US). The gating strategy was previously published [24].

#### 2.2.5 Detection of sexually transmitted infections (STIs) and vaginal microbes

Genital swabs were used to detect common STI pathogens and vaginal microbes, as previously described (Table S3) [29]. Multiplex PCR amplification was conducted to detect STI pathogens using the Fast‐track Diagnostics STD9 detection kit, as outlined in the manufacturer's protocol. Common vaginal microbes were detected using Applied Biosystems™ TaqMan^®^ assays [29]. All reactions were conducted using the Applied Biosystems 7500 RT‐PCR machine (Thermo Fisher Scientific). Data on STI detection in vaginal specimens were available for all visits (n = 648), whereas data on bacterial vaginosis (BV)‐associated bacteria were generated for all visits except baseline (n = 515; Figure S1). BV was diagnosed using Nugent scoring by Gram stain microscopy [30].

#### 2.2.6 Statistical considerations

Statistical analyses were performed using GraphPad Prism version 8.4.3 (GraphPad Software, San Diego, CA, USA), STATA version 15.0 (StataCorp., College Station, TX, USA) and SAS version 9.4 (SAS Institute Inc., Cary, NC, USA). Continuous variables and proportions were compared at baseline using the Kruskal–Wallis test and the chi‐square or Fisher’s exact test respectively. Longitudinal analyses included one model with YcDNA concentrations as the main exposure variable and a complementary model with PSA/YcDNA categories as the main exposure variable. CVL specimens were classified in terms of PSA and YcDNA detection as PSA−YcDNA−, PSA+YcDNA+ and PSA−YcDNA+. The Mann–Whitney test was used to determine whether YcDNA concentrations differed significantly between PSA+YcDNA+ and PSA−YcDNA+ specimens at baseline. Receiver operating characteristic (ROC) curve analysis was used to plot the lowest cut‐off YcDNA concentration that predicted PSA positivity. Linear regression models were used to determine the impact of PSA+YcDNA+ and PSA‐YcDNA+ events on MMP/TIMP concentrations compared to PSA−YcDNA− events at baseline. Spearman’s Rank correlation was used to determine the relationship between concentrations of YcDNA and MMPs/TIMPs at baseline. Linear mixed models (LMMs) adjusting for repeated measures were used to assess the impact of PSA+YcDNA+ and PSA−YcDNA+ events on cytokine concentrations and immune cell frequencies compared to PSA−YcDNA− events over multiple visits. Generalized estimating equation (GEE) models using a logit link were used to compare microbe presence between PSA+YcDNA+, PSA−YcDNA+ and PSA−YcDNA− groups over multiple visits. Multivariable LMMs adjusted for the contribution of study arm (CAPRISA or family planning clinics), time in the study, Nugent Score, participant age, STIs, the frequency of vaginal sex in the past month, and genital inflammation status [4, 28]. The Benjamini–Hochberg method was used to calculate the false discovery rate (FDR).

## 3 Results

### 3.1 Study population

Baseline clinical and demographic data are reported for 137/152 women with both PSA and YcDNA data available (Table 1). The study participants’ overall median age was 28 years (interquartile range (IQR) 25 to 33 years). Semen exposure was determined by YcDNA detection (YcDNA+), with PSA detection further stratifying participant groups into semen exposure within 48 hours of sampling (PSA+YcDNA+) and between three and fifteen days of sampling (PSA−YcDNA+). At baseline, 28% (38/137) of women were PSA+YcDNA+, 14% (19/137) were PSA−YcDNA+ and 58% (80/137) were PSA−YcDNA−, suggesting no semen exposure within 15 days of sampling. Semen exposure was associated with how often women saw their partners (*p* = 0.038), the frequency of vaginal sex in the past month (*p* = 0.006), Herpes simplex virus (HSV)‐2 detection (*p* = 0.047), current gonorrhoea infection (*p* = 0.018) and Nugent Score (*p* = 0.003). Higher median Nugent Scores were specifically driven by semen exposure within 48 hours compared to no semen exposure (median 3 (IQR 1 to 7) vs. median 1 (IQR 0 to 3), respectively, *p* = 0.003). Furthermore, women with evidence of semen exposure within three to fifteen days reported more frequent acts of vaginal sex in the last month than women with no evidence of semen exposure (median 8 (IQR 4 to 10) vs. median 4 (IQR 2 to 6), respectively, *p* = 0.017).

**Table 1:** Baseline characteristics of the study participants grouped by the timing of semen exposure

Characteristics	Level	No semen exposure	Semen exposure		*p*‐value
		PSA−YcDNA− (n = 80)	Within 48 hours PSA+YcDNA+ (n = 38)	Within three to fifteen days PSA−YcDNA+ (n = 19)	
Median age (IQR)		28 (25 to 30)	29 (25 to 35)	29 (24 to 33)	0.543
Study arm [% (n)]	Intervention	43.8 (35)	55.3 (21)	42.1 (8)	0.458
	Control	56.3 (45)	44.7 (17)	57.9 (11)	
Age of sexual debut (year)	Median (IQR)	18 (17 to 19)	18 (16 to 20)	17 (16 to 18)	0.120
Age at menarche	Median (IQR)	14 (13 to 16)	15 (13 to 16)	14 (13 to 16)	0.917
Number of lifetime partners	Median (IQR)	3 (2 to 4)	2 (1 to 3)	2 (1 to 4)	0.184
Relationship status [% (n)]	Married	10.0 (8)	28.9 (11)	15.8 (3)	0.103
	Stable partner	88.8 (71)	68.4 (26)	84.2 (16)	
	Casual partner	1.3 (1)	2.6 (1)	0	
How often you see your partner [% (n)]	Daily	19.0 (15/79)	34.2 (13)	42.1 (8)	0.038*
	Weekly	40.5 (32/79)	47.4 (18)	47.4 (9)	
	Monthly	36.7 (29/79)	18.4 (7)	5.3 (1)	
	<Monthly	3.8 (3/79)	0	5.3 (1)	
Male condom use [% (n)]	Always	42.5 (34)	26.3 (10)	36.8 (7)	0.345
	Sometimes	47.5 (38)	55.3 (21)	42.1 (8)	
	Never	10.0 (8)	18.4 (7)	21.1 (4)	
Vaginal sex acts in the last 30 days	Median (IQR)	4 (2 to 6)	4 (3 to 11)	8 (4 to 10)^b^	0.006*
Partner circumcision [% (n)]	Yes	36.8 (25/68)	24.2 (8/33)	33.3 (6/18)	0.709
	No	60.3 (41/68)	72.7 (24/33)	66.7 (12/18)	
Partner HIV status [% (n)]	Positive	1.3 (1)	2.6 (1)	5.3 (1)	0.565
	Negative	62.5 (50)	73.7 (28)	63.2 (12)	
	Unknown	36.3 (29)	23.7 (9)	31.6 (6)	
Herpes simplex virus 2 [% (n)]		89.6 (69/77)	92.1 (35)	73.7 (14)	0.047*
Human papillomavirus [% (n)]		50.0 (40)	50.0 (19)	47.4 (9)	0.978
*Neisseria gonorrhoeae* [% (n)]		0	10.5 (4)	5.3 (1)	0.018*
*Chlamydia trachomatis* [% (n)]		7.7 (6/78)	10.5 (4)	0	0.356
*Trichomonas vaginalis* [% (n)]		5.1 (4/78)	2.6 (1)	5.3 (1)	0.815
*Mycoplasma genitalium* [% (n)]		3.8 (3/78)	5.3 (2)	5.3 (1)	0.925
Bacterial vaginosis (Nugent Score)	Median (IQR)	1 (0 to 3)	3 (1 to 7)^a^	1 (0 to 6)	0.003*
Negative [% (n)]	0 to 3	84.2 (64/76)	57.9 (22)	68.4 (13)	0.004*
Intermediate [% (n)]	4 to 6	9.2 (7/76)	7.9 (3)	15.8 (3)	
BV [% (n)]	7 to 10	6.6 (5/76)	34.2 (13)	15.8 (3)	

The chi‐square and Fisher’s exact tests were used to compare proportions between the groups as deemed appropriate. Continuous data were assessed by Kruskal–Wallis tests to compare differences between no semen exposure (PSA−YcDNA−), semen exposure within 48 hours (PSA+YcDNA+) and semen exposure within three to fifteen days (PSA−YcDNA+), Dunn’s post‐testing was applied to adjust for multiple comparisons. Significant differences between no semen exposure and semen exposure within 48 hours are indicated by (a), whereas differences between no semen exposure and exposure within three to fifteen days are indicated by (b). Significant *p*‐values (*p* < 0.05) are indicated by (*) and bold font. BV, bacterial vaginosis; HIV, human immunodeficiency virus; IQR, interquartile range; PSA, prostate‐specific antigen; YcDNA, Y‐chromosome DNA.

### 3.2 Higher cervicovaginal YcDNA concentrations reflect more recent semen exposure

**Figure 1:**
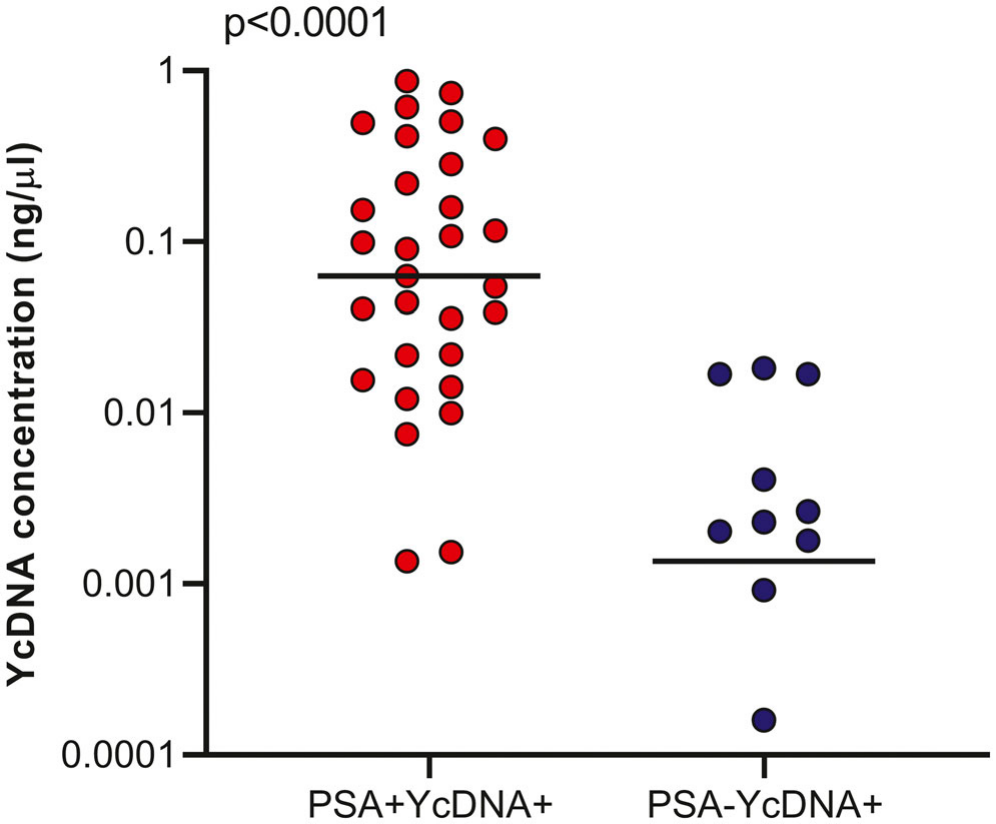
Relationship between YcDNA concentrations and timing of semen exposure prior to cervicovaginal sampling. The Mann–Whitney U test was used to compare YcDNA concentrations between women with evidence of semen exposure within 48 hours (PSA+YcDNA+; n = 30) and three to fifteen days (PSA−YcDNA+; n = 10) at baseline. PSA, prostate‐specific antigen; YcDNA, Y‐chromosome DNA.

In women with evidence of condomless sex, we assessed the relationship between YcDNA concentrations and the timing of semen exposure. At baseline, only 70% (40/57) of YcDNA+ specimens (i.e. PSA+YcDNA+ and PSA−YcDNA+ specimens) had a yield sufficient for quantitation. Women with detectable genital PSA (PSA+YcDNA+; n = 30) had significantly higher YcDNA concentrations than women without (PSA−YcDNA+; n = 10; median 0.077 ng/µL (IQR 0.020 to 0.314) vs. 0.002 ng/µL (0.002 to 0.017) respectively; *p* < 0.0001; Figure 1).

Since higher YcDNA concentrations were observed soonest after semen exposure, it suggests a potential for a threshold YcDNA concentration to serve as a proxy for the timing of semen exposure before genital sampling. ROC curve analysis suggests a YcDNA concentration cutoff of 0.005 ng/µL predicts PSA positivity with the greatest sensitivity [87.4%, confidence interval (CI) 81.2, 93.6] and with a specificity of 62.3% (CI 50.9, 73.8; Table S4; Figure S2).

### 3.3 Semen‐associated alterations to cervicovaginal cytokines

**Figure 2:**
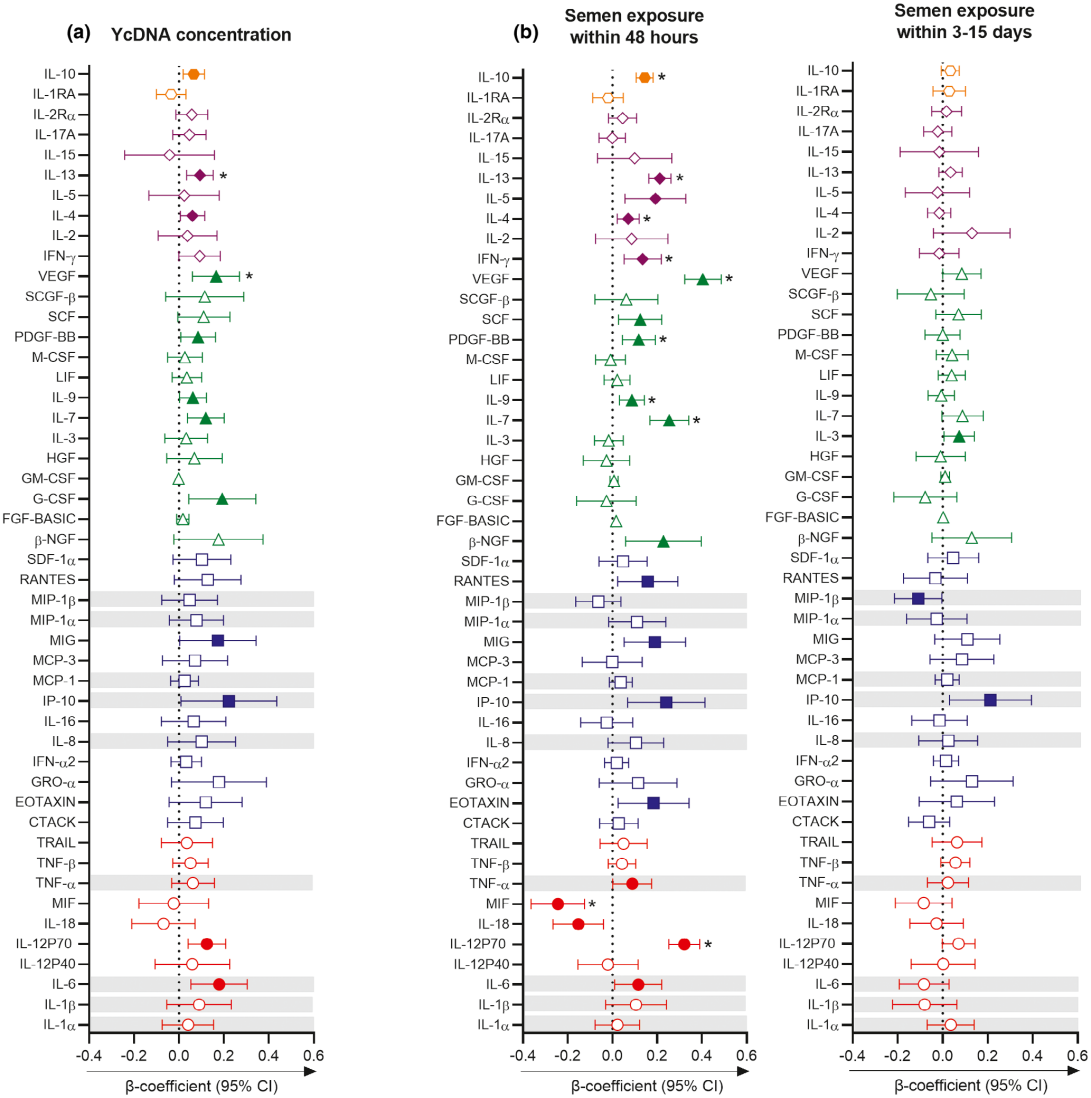
Associations between semen exposure and cervicovaginal cytokine concentrations. Multivariable linear mixed models controlling for study arm, time in study, participant age, the frequency of vaginal sex in the past month, sexually transmitted infections (*Chlamydia trachomatis*, *Neisseria gonorrhoeae*, *Trichomonas vaginalis* and *Mycoplasma genitalium*) and Nugent Score were used to determine the association between semen exposure and vaginal cytokine concentrations over multiple visits. **(a)** Associations between YcDNA concentrations and cervicovaginal cytokine concentrations over multiple visits (n = 167 genital specimens). **(b)** Longitudinal comparison of cytokine concentrations between semen exposure within 48 hours (PSA+YcDNA+ specimens; n = 124) and three to fifteen days (PSA−YcDNA+ specimens; n = 93) relative to no semen exposure (PSA−YcDNA− specimens; n = 433). Cytokines are ordered according to their functions: pro‐inflammatory (red circles), chemotactic (blue squares), growth/haematopoiesis (green triangles), adaptive response (purple diamonds) and regulatory (orange hexagons) cytokines. Grey shadings represent the cytokines previously associated with genital inflammation and in demonstrating its association with HIV risk in this cohort [4, 28]. β‐coefficients are depicted by shapes and error bars indicate the 95% CI. Filled shapes indicate significant *p*‐values (*p* < 0.05), and significance after FDR adjustment is indicated by (*). Table S1 contains a list of abbreviations for the 48 cytokines measured in this study. CI, confidence interval; YcDNA, Y‐chromosome DNA.

Since semen’s impact on the FGT is likely short‐lived [1, 22], we hypothesized that higher YcDNA concentrations and semen exposure within 48 hours would be associated with greater alterations in cervicovaginal cytokines (Table S1). Multivariable LMMs were used to compare YcDNA concentrations and cytokine levels over multiple visits (n = 167 genital specimens). Higher YcDNA concentrations were associated with elevated concentrations of 12/48 cytokines (Figure 2a). The strongest associations were observed between higher YcDNA concentrations and IL‐13 (β = 0.093; CI 0.034, 0.152; *p* = 0.002) and VEGF (β = 0.165; CI 0.060, 0.269; *p* = 0.002), both maintaining statistical significance after FDR adjustment.

Cytokine concentrations were also compared between PSA+YcDNA+ specimens (n = 124) and PSA−YcDNA+ specimens (n = 93) relative to PSA−YcDNA− specimens (n = 433) over multiple visits. PSA+YcDNA+ events were significantly associated with increases in 18/48 cytokines and with reductions in two cytokines relative to PSA−YcDNA− events (Figure 2b). The strongest associations were observed with IL‐12p70 (β = 0.321; CI 0.252, 0.390), IL‐13 (β = 0.211; CI 0.162, 0.261), IL‐7 (β = 0.254; CI 0.167, 0.341), VEGF (β = 0.404; CI 0.323, 0.486), MIF (β = −0.244; CI −0.364, −0.125) and IL‐10 concentrations (β = 0.144; CI 0.106, 0.182; all *p* < 0.001). In comparison, PSA−YcDNA+ specimens only had increased concentrations of IP‐10 (β = 0.212; CI 0.030, 0.395; *p* = 0.023) and IL‐3 (β = 0.074; CI 0.006, 0.141; *p* = 0.034).

### 3.4 Semen‐associated alterations to barrier‐related proteins

**Figure 3:**
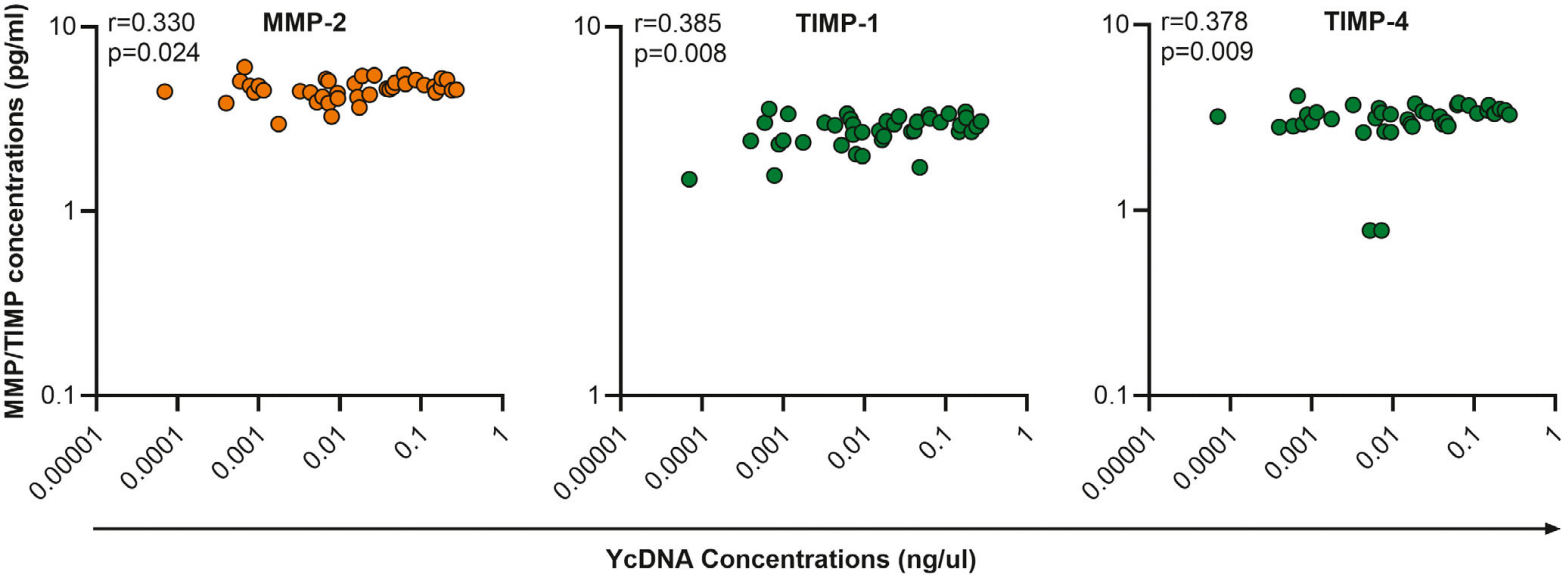
Correlations between YcDNA concentrations and MMP/TIMP concentrations at baseline. Spearman’s Rank correlations were used to determine the relationship between YcDNA concentrations and MMP/TIMP concentrations at baseline (n = 40). Orange circles represent MMPs, and green circles represent their inhibitors (TIMPs). MMP, matrix metalloproteinase; TIMP, tissue inhibitors of metalloproteinases; YcDNA, Y‐chromosome DNA.

MMPs and their regulators, TIMPs are involved in the remodelling of the extracellular matrix. Increased expression of MMPs is associated with elevated genital cytokines, epithelial barrier disruption and HIV transmigration [5, 31]. Therefore, we compared cervicovaginal YcDNA concentrations with MMP/TIMP concentrations at baseline (n = 40). Moderate positive correlations were observed between YcDNA concentrations and the levels of MMP‐2 (r = 0.330; CI 0.039, 0.570; *p* = 0.024), TIMP‐1 (r = 0.385; CI 0.102, 0.611; *p* = 0.008) and TIMP‐4 (r = 0.378; CI 0.093, 0.605; *p* = 0.009; Figure 3).

**Figure 4:**
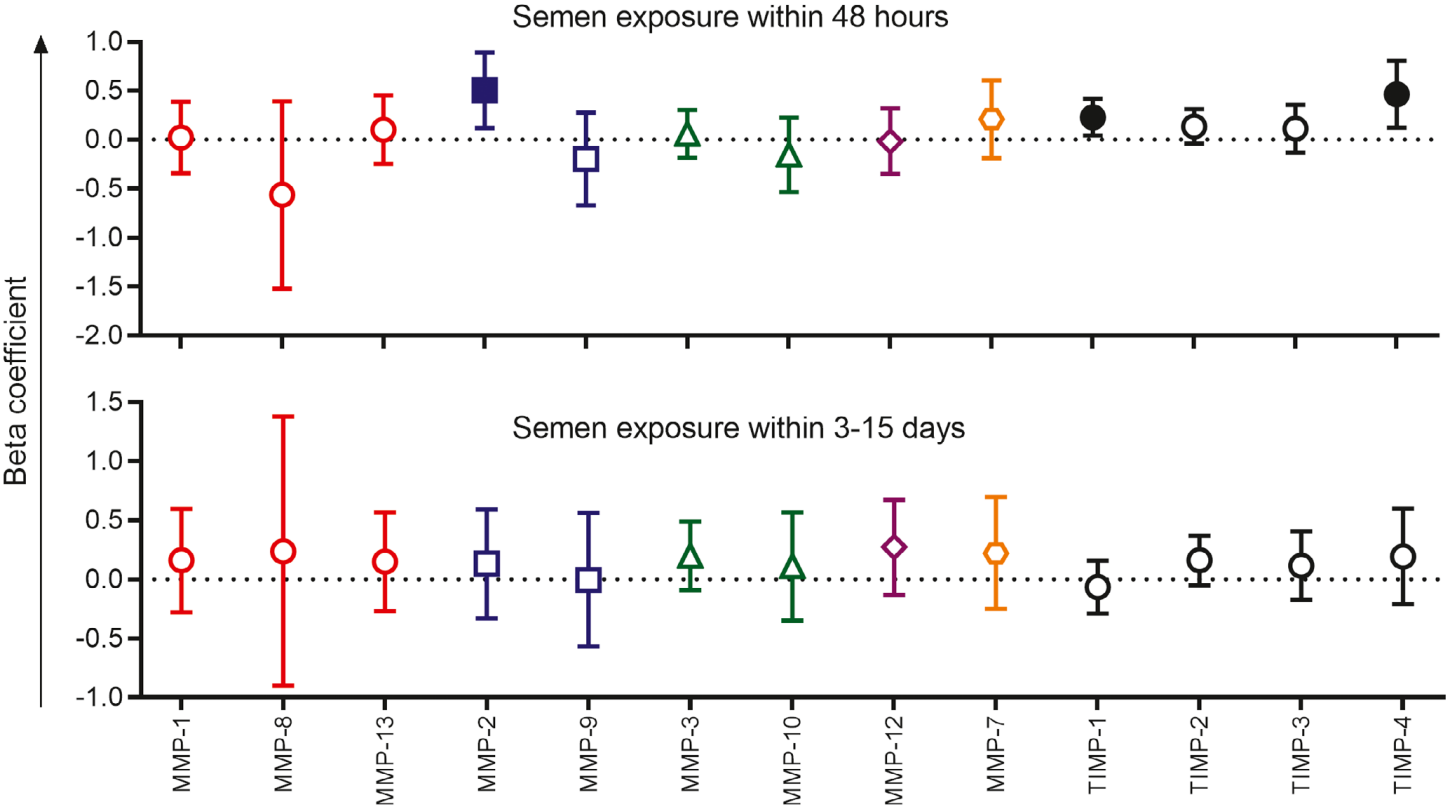
Associations between MMP/TIMP concentrations and timing of semen exposure at baseline. Multivariable linear regression models were used to compare MMP/TIMP concentrations at baseline between women with evidence of semen exposure within 48 hours (n = 37) and three to fifteen days (n = 19) relative to those with no semen exposure within the 15 days (n = 80) before genital sampling. Models were adjusted for age, sexually transmitted infections (*Chlamydia trachomatis*, *Neisseria gonorrhoeae*, *Trichomonas vaginalis* and *Mycoplasma genitalium*), Nugent Score, the frequency of vaginal sex in the past month, study arm and inflammation status. β‐coefficients are depicted by shapes and error bars indicate the 95% confidence intervals. Filled shapes indicate significant *p*‐values (*p* < 0.05). MMPs are grouped according to their function: collagenases (red circles), gelatinases (blue squares), stromelysins (green triangles), macrophage elastase (purple diamond), matrilysin (orange hexagon) and TIMPs are represented by black circles. MMP, matrix metalloproteinase; TIMP, tissue inhibitors of metalloproteinases.

Similarly, PSA+YcDNA+ CVLs (n = 37) had higher concentrations of MMP‐2 (β = 0.506; CI 0.119, 0.892; *p* = 0.011), TIMP‐1 (β = 0.230; CI 0.043, 0.417; *p* = 0.016) and TIMP‐4 (β = 0.466; CI 0.125, 0.808; *p* = 0.008; Figure 4). No significant associations were observed with semen exposure within three to fifteen days (PSA−YcDNA+ CVLs; n = 19).

### 3.5 Semen‐associated alterations to endocervical immune cell frequencies

**Figure 5:**
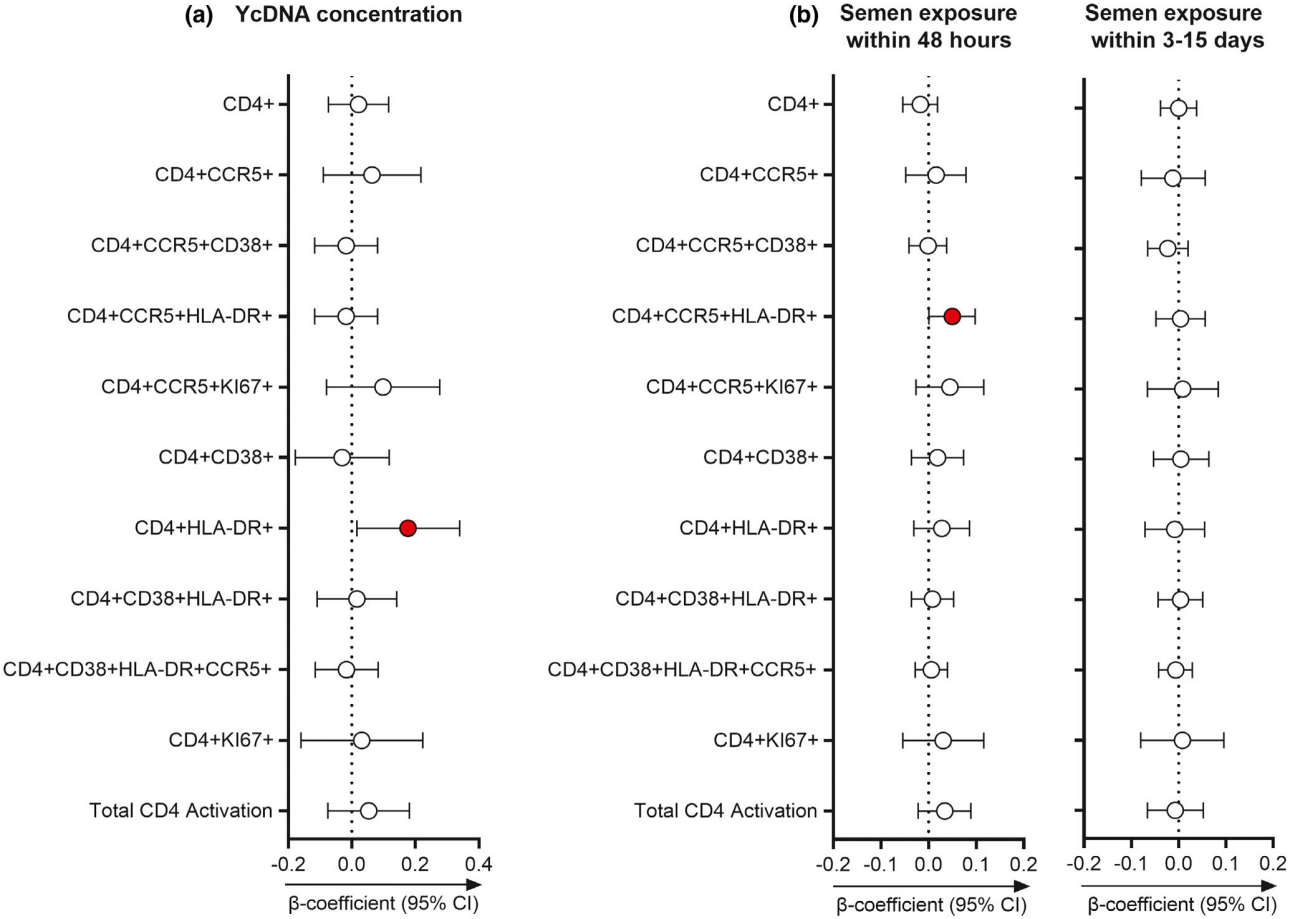
Associations between semen exposure and endocervical immune cell frequencies. Linear mixed models adjusting for the time in study, study arm, participant age, the frequency of vaginal sex in the past month, sexually transmitted infections (*Chlamydia trachomatis*, *Neisseria gonorrhoeae*, *Trichomonas vaginalis* and *Mycoplasma genitalium*), inflammation status and Nugent Score were used to determine the relationship between semen exposure and endocervical T cell frequencies over multiple visits. **(a)** Associations between YcDNA concentrations and immune cell frequencies over multiple visits (n = 145 genital specimens). **(b)** Longitudinal comparison of immune cell frequencies between semen exposure within 48 hours (PSA+YcDNA+ specimens; n = 108) and three to fifteen days (PSA−YcDNA+ specimens; n = 85) relative to no semen exposure (PSA−YcDNA− specimens; n = 375). β‐coefficients are depicted by circles and error bars indicate the 95% CI. Red filled circles indicate significant *p*‐values (*p* < 0.05). Total CD4 activation refers to cells expressing CCR5, HLA‐DR, and/or CD38. CI, confidence interval; YcDNA, Y‐chromosome DNA.

Since HIV must gain access to local target cells for infection, we investigated whether YcDNA concentrations and the timing of semen exposure were associated with alterations in endocervical immune cell frequencies. In multivariable LMMs, higher YcDNA concentrations were associated with increased frequencies of the activated CD4+HLA‐DR+ T cell populations (n = 145 genital specimens; β = 0.177; CI 0.016, 0.339; *p* = 0.032; Figure 5a).

Additionally, semen exposure within 48 hours (n = 108 genital specimens) was associated with higher frequencies of activated HIV target cells (CD4+CCR5+HLA‐DR+; β = 0.050; CI 0.001, 0.098; *p* = 0.046; Figure 5b). No significant associations were observed with semen exposure within three to fifteen days (n = 85 genital specimens).

### 3.6 Semen‐associated alterations to vaginal microbes

**Figure 6:**
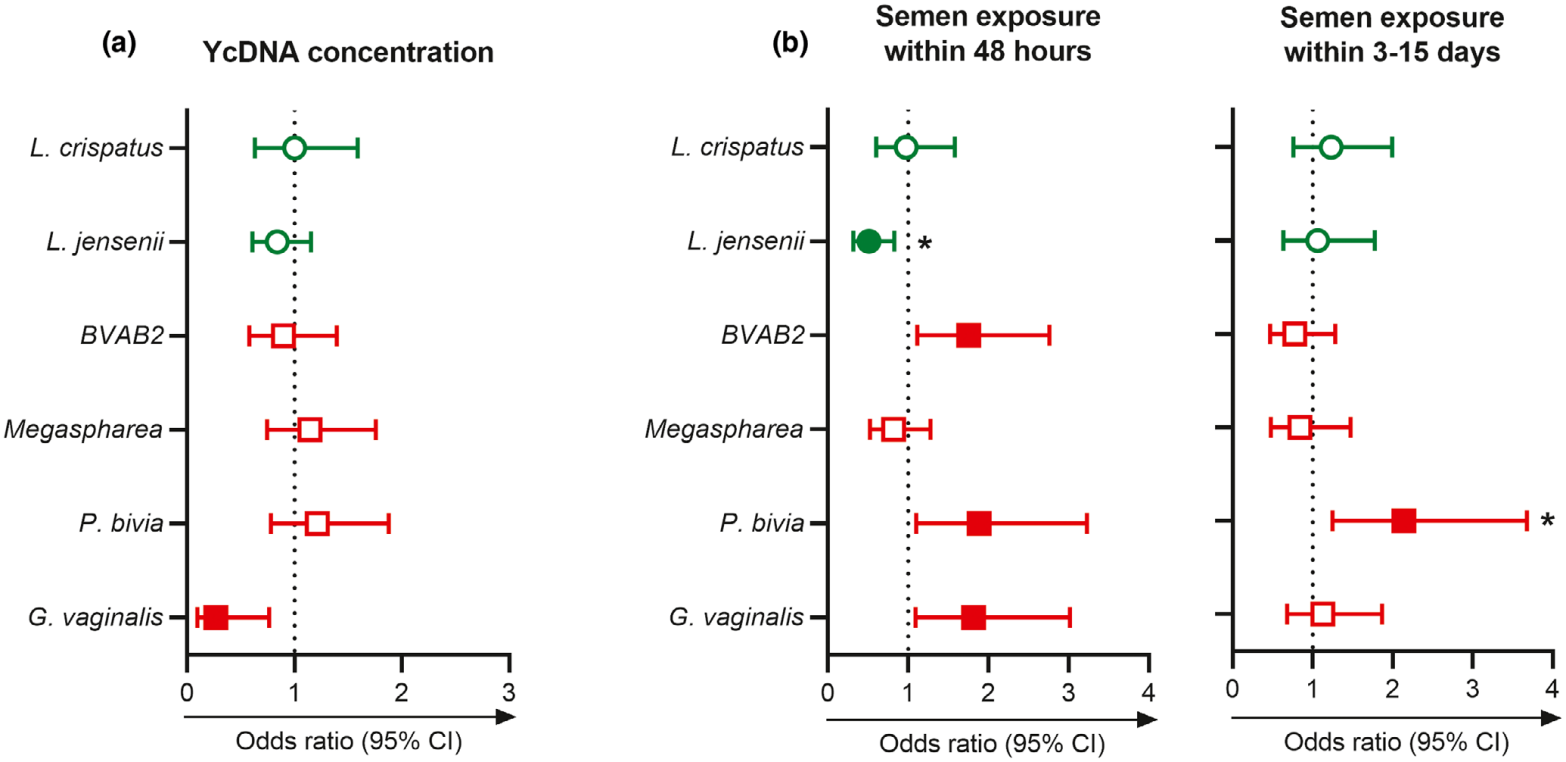
Semen‐associated alterations to post‐coital vaginal microbes. GEE models were used to determine the association between semen exposure and microbe presence over multiple visits. **(a)** Comparisons between YcDNA concentrations and vaginal microbe presence (n = 133 genital specimens). **(b)** Microbe presence was compared between semen exposure within 48 hours (PSA+YcDNA+ specimens; n = 87) and three to fifteen days (PSA−YcDNA+ specimens; n = 74) relative to no semen exposure within 15 days (PSA−YcDNA− specimens; n = 354) before genital sampling. Models adjusted for participant age, the frequency of vaginal sex in the past month, sexually transmitted infections (*Chlamydia trachomatis*, *Neisseria gonorrhoeae*, *Trichomonas vaginalis* and *Mycoplasma genitalium*), inflammation status, time in the study and study arm. Odds ratios are depicted by shapes and error bars indicate the 95% CI. Filled shapes indicate significant *p*‐values (*p* < 0.05), and (*) indicates significance after FDR adjustment. BVAB2, bacterial vaginosis‐associated bacterium 2; CI, confidence interval; *G. vaginalis*, *Gardnerella vaginalis; L. crispatus*, *Lactobacillus crispatus; L. jensenii*, *Lactobacillus jensenii; P. bivia*, *Prevotella bivia;* YcDNA, Y‐chromosome DNA.

Since vaginal microbial diversity is associated with HIV infection in women [6, 7], we examined the contribution of YcDNA concentrations and the timing of semen exposure on vaginal microbe detection over multiple visits [6, 7]. In adjusted GEE models, higher YcDNA concentrations were associated with reduced detection of *Gardnerella vaginalis* in vaginal specimens (n = 133; odds ratio (OR) = 0.269; CI 0.095, 0.764; *p* = 0.014; Figure 6a).

Semen exposure within 48 hours (n = 87 genital specimens) was associated with more frequent detection of BVAB2 (OR = 1.755; CI 1.116, 2.760; *p* = 0.015), *P*. *bivia* (OR = 1.886; CI 1.102, 3.228; *p* = 0.021), *G. vaginalis* (OR = 1.815; CI 1.093, 3.015; *p* = 0.021) and reduced detection of *Lactobacillus jensenii* (OR = 0.515; CI 0.319, 0.831; *p* = 0.007; Figure 6b) over multiple visits. Semen exposure within three to fifteen days was only associated with increased detection of *P*. *bivia* (n = 74 genital specimens; OR = 2.142; CI 1.248, 3.676; *p* = 0.006).

## 4 Discussion

**Figure 7:**
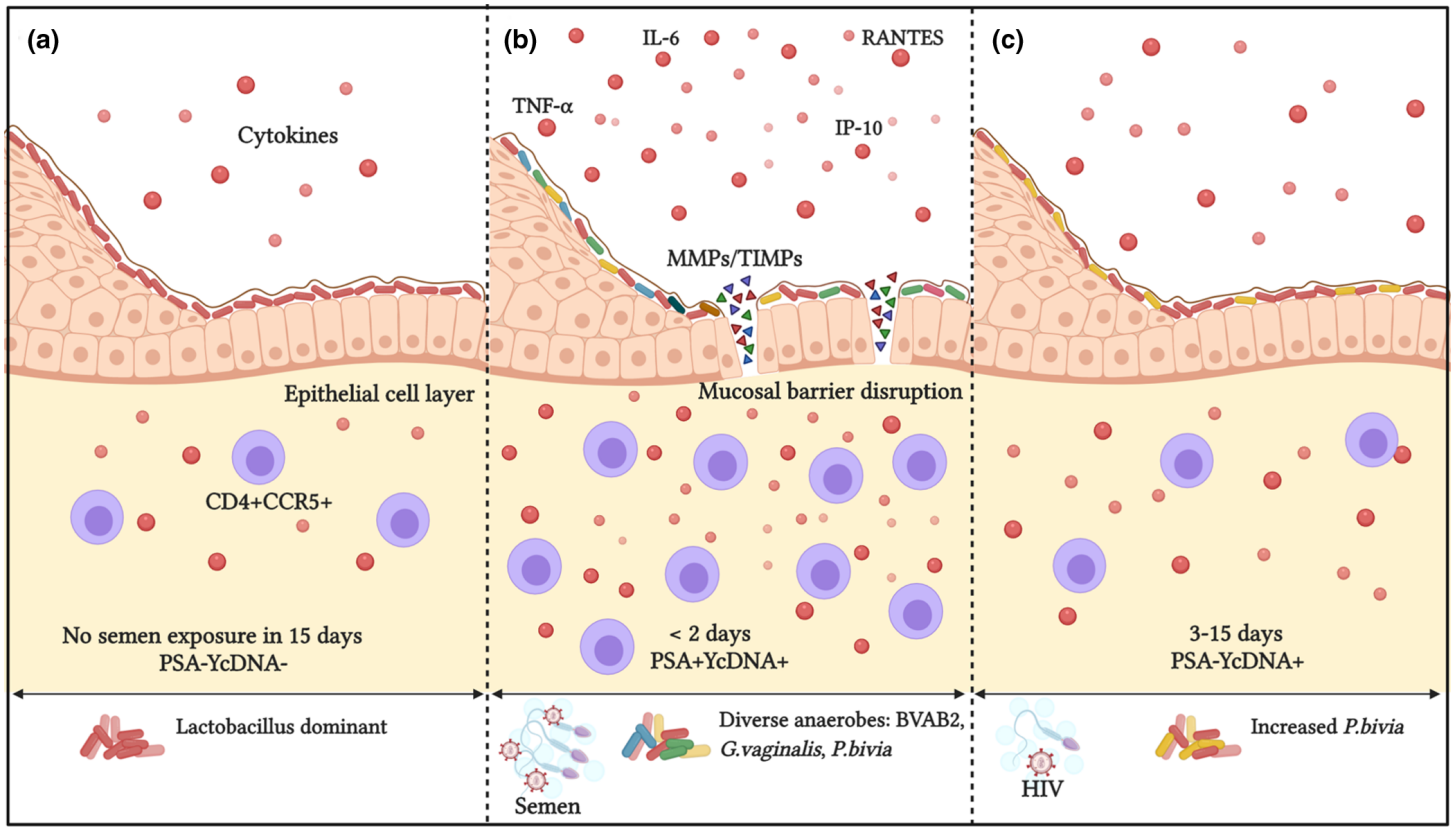
Graphical representation of semen‐associated alterations at the female genital mucosa. **(a)** An optimal vaginal environment in the absence of semen exposure. Here, the vaginal microbiome is dominated by Lactobacillus, few cytokines and immune cells are present at the FGT, and the vaginal epithelial barrier is intact. **(b)** In this study, recent semen exposure <2 days before genital sampling and higher YcDNA concentrations were associated with elevated concentrations of several cytokines, MMPs/TIMPs, BV‐associated microbes and HIV target cell recruitment at the female genital mucosa compared to no semen exposure within 15 days. **(c)** In comparison, semen exposure within three to fifteen days was only associated with moderate alterations in cervicovaginal cytokine concentrations and increased detection of *P*. *bivia* compared to no semen exposure within 15 days. BVAB2, bacterial vaginosis‐associated bacterium 2; *G. vaginalis*, *Gardnerella vaginalis;* HIV, human immunodeficiency virus; IL‐6, interleukin‐6; IP‐10, interferon gamma‐induced protein‐10; MMP, matrix metalloproteinase; *P*. *bivia*, *Prevotella bivia;* PSA, prostate‐specific antigen; RANTES, regulated on activation, normal T cell expressed and secreted; TIMP, tissue inhibitor of metalloproteinases; TNF‐α, tumour necrosis factor‐alpha; YcDNA, Y‐chromosome DNA.

We investigated the contribution of recent semen exposure to the inflammatory environment linked to HIV risk in women. PSA detection and higher YcDNA concentrations, both suggesting more recent semen exposure, were associated with elevated cervicovaginal cytokines, barrier‐related proteins, increased detection of BV‐associated microbes and higher HIV target cell frequencies in vaginal specimens (Figure 7). These findings suggest that semen‐associated immune responses at the FGT are transient and highlight the importance of considering the timing of genital sampling after condomless sex.

At baseline, more than a third of the women had detectable YcDNA, indicating semen exposure within a range of 15 days before genital sampling. The timing of semen exposure was specifically related to the number of vaginal sex acts reported and the BV Nugent Score. Semen exposure within three to fifteen days allowed for a longer range of semen detection and was therefore associated with a greater number of coital episodes in the last month. Semen exposure within 48 hours was associated with higher Nugent Scores, suggesting that condomless sex is linked to a short‐term increased presence of BV‐associated microbes at the FGT.

We assessed the relationship between YcDNA quantities and the timing of semen exposure. Concentrations of YcDNA at the FGT may indicate the amount of seminal proteins and sperm count in the ejaculate of the male partner during condomless sex. These data demonstrated that YcDNA concentrations are also related to the timing of semen exposure since PSA detection was associated with higher YcDNA concentrations. The Quantifiler™ Trio DNA Quantification Kit used in this study correctly predicted PSA positivity in vaginal specimens with even minimal levels of YcDNA. YcDNA concentrations could be useful in studies requiring the use of a single semen biomarker, which also indicates the timing of semen exposure. Further studies are needed to confirm the feasibility of using YcDNA concentrations to determine more recent semen exposure (i.e. PSA detection) before genital sampling.

Semen exposure within 48 hours and higher YcDNA concentrations were associated with raised levels of several cytokines, whereas semen exposure within three to fifteen days was associated with increased concentrations of only two cytokines (IL‐3 and IP‐10). Although transient, elevated concentrations of IL‐6, TNF‐α, IP‐10, RANTES and IL‐10 associated with more recent semen exposure have also been related to increased HIV risk in women [4, 26, 32]. Of particular importance are the chemokines, with raised IP‐10 independently associated with HIV seroconversion and T‐cell recruitment [4, 33], and RANTES, a CCR5 ligand, involved in both the blocking of HIV binding to CCR5 on target cells and the recruitment of these target cells to the FGT [34]. Recent semen exposure was also associated with increased concentrations of MMP‐2, TIMP‐1 and TIMP‐4. Increased MMPs/TIMPs may signify wound healing since microabrasions are introduced at the FGT during coitus [35] and may also impact HIV risk in women through reduced mucosal barrier integrity and/or target cell recruitment [5].

Our findings confirmed that more recent semen exposure was also associated with increased frequencies of activated endocervical CD4 T cells (CD4+HLA‐DR+ and CD4+CCR5+HLA‐DR). Studies suggest that semen exposure at the FGT is associated with an initial pro‐inflammatory response resulting in leucocyte recruitment to remove excess and abnormal sperm [1, 36]. This may explain the increase in CD4+HLA‐DR+ T cell frequencies in response to higher YcDNA concentrations observed in this study, which may also indicate higher sperm counts. Others have reported that this inflammatory response dissipates within 48 to 72 hours, and a regulatory T cell immune response is mounted to facilitate conception [2, 3, 37]. This may also explain why semen exposure within three to fifteen days was not related to immune cell alterations in this study. Activated endocervical CD4 T cells are putative HIV target cells, and increased frequencies of these cells may promote heterosexual transmission of HIV from an infected male partner.

Pathogens, STIs and commensal microbes present in semen and at the male genital tract [11, 12, 38, 39] are transferred to the FGT during condomless sex and could alter the vaginal microbial composition [11, 40]. Surprisingly, higher concentrations of cervicovaginal YcDNA were associated with reduced detection of *G. vaginalis*. Further studies are required to determine reasons for this association, for example, the contribution of semen‐derived antimicrobial activities, the impact of semen on *G. vaginalis* growth dynamics, etc. Conversely, semen exposure within 48 hours was associated with increased detection of several BV‐associated microbes (BVAB2, *P*. *bivia* and *G. vaginalis*) and reduced detection of *L*. *jensenii*, *whereas* women exposed to semen within three to fifteen days only had an increased detection of *P*. *bivia* in vaginal specimens. Studies have demonstrated that a diverse vaginal microbiome with increased Prevotella and reduced *Lactobacillus* *crispatus* is associated with a higher risk of HIV acquisition among women [6, 7]. This is due to the ability of BV‐associated microbes to induce cytokine production and the recruitment of activated CD4+ T cells, both of which were observed in this study [6, 7].

A strength of this study was the use of recent semen biomarkers and the wealth of immune and microbial data to reliably assess the effect of semen exposure on the female genital mucosa over multiple visits. To our knowledge, this is the first study to assess the impact of YcDNA concentrations and the timing of semen exposure on markers of genital inflammation related to HIV risk in women. Given that semen itself contains several endogenously produced cytokines and CD4+ T cells from the male partner [10, 22, 41, 42], a limitation of this study was the inability to determine whether the elevated immune responses detected were from semen itself or an immune response elicited at the FGT. However, Chen *et al*. [43], demonstrated that endometrial epithelial cells and stromal fibroblasts treated with seminal plasma had raised levels of several cytokines after adjusting for endogenous seminal plasma cytokines. Similar to our findings, seminal plasma exposure was associated with elevated G‐CSF, IL‐6, TNF‐α and VEGF among others. This study distinguished cytokines produced by the FGT from endogenous seminal plasma cytokines and suggests that the immune markers detected here were mounted by the FGT and not due to residual seminal components.

## 5 Conclusions

This longitudinal study demonstrates that semen exposure induces a transient pro‐inflammatory immune response at the FGT which may significantly impact HIV risk in women. Here, PSA detection and higher concentrations of YcDNA, both indicative of recent semen exposure, were associated with increases in cervicovaginal cytokines and barrier‐related proteins, recruitment, and activation of endocervical CD4 T cells and alterations in vaginal microbes. These findings emphasize the need for studies of genital mucosal immunity to STIs such as HIV to consider the contribution of semen exposure to the FGT immune and microbial environments, preferably using a biomarker of recent semen exposure. This study also highlights the importance of consistent condom use, particularly in settings where women are at increased risk of acquiring HIV.

## Competing interests

The authors declare that they have no competing interests.

## Authors’ contributions

J.J., S.N. and L.J.P.L. contributed to the conception and design of the study. J.J., L.J.P.L., A.M., M.F. and R.S. performed the experiments. J.J., L.J.P.L. and F.O. analysed and interpreted the data. J.J. and L.J.P.L. wrote the manuscript. All authors have read and approved the final manuscript.

## Supporting information


**Table S1**. List of cytokines measured in cervicovaginal lavage supernatant specimens
**Table S2**. Flow cytometry information on the antibody clones, fluorophores, and suppliers
**Table S3**. List of common STI pathogens and other vaginal microbes measured in vulvovaginal swabs
**Table S4**. Sensitivity and specificity for the YcDNA concentration cutoff value of 0.005 ng/µL
**Figure S1**. Graphical representation of the data available at baseline and longitudinally for CAPRISA 008 study participants.
**Figure S2**. ROC curve for all YcDNA concentration cutoff values.
