# Supplementary material for: Transient association between semen exposure and biomarkers of genital inflammation in South African women at risk of HIV infection
Source: J Int AIDS Soc. 2021 Jun 24;24(6):e25766. doi: 10.1002/jia2.25766 (PMC8223121; doi:10.1002/jia2.25766)
Supplement: Supplementary file 1 — Table S1. List of cytokines measured in cervicovaginal lavage supernatant specimens Table S2. Flow cytometry information on the antibody clones, fluorophores, and suppliers Table S3. List of common STI pathogens and other vaginal microbes measured in vulvovaginal swabs Table S4. Sensitivity and specificity for the YcDNA concentration cutoff value of 0.005 ng/µL Figure S1. Graphical representation of the data available at baseline and longitudinally for CAPRISA 008 study participants. Figure S2. ROC curve for all YcDNA concentration cutoff values. [file JIA2-24-e25766-s001.docx]

**Table S1.** List of cytokines measured in cervicovaginal lavage supernatant specimens.

| **Abbreviation** | **Cytokine Name** |
| --- | --- |
| IL-1α | interleukin-1 alpha |
| IL-1β | interleukin-1 beta |
| IL-2 | interleukin-2 |
| IL-3 | interleukin-3 |
| IL-4 | interleukin-4 |
| IL-5 | interleukin-5 |
| IL-6 | interleukin-6 |
| IL-7 | interleukin-7 |
| IL-8 | interleukin-8 |
| IL-9 | interleukin-9 |
| IL-10 | interleukin-10 |
| IL-12p40 | interleukin-12 p40 |
| IL-12p70 | interleukin-12 p70 |
| IL-13 | interleukin-13 |
| IL-15 | interleukin-15 |
| IL-16 | interleukin-16 |
| IL-17 | interleukin-17 |
| IL-18 | interleukin-18 |
| IL-1RA | interleukin-1 receptor antagonist |
| IL-2Rα | interleukin-2 receptor alpha |
| CTACK | cutaneous T cell attracting chemokine |
| GRO-α | growth-related oncogene alpha |
| HGF | hepatocyte growth factor |
| IFN-γ | Interferon-gamma |
| IFN-α2 | interferon alpha-2 |
| LIF | leukaemia inhibitory factor |
| MCP-3 | monocyte chemotactic protein-3 |
| MIF | macrophage migration inhibitory factor |
| MIG | monokine induced by gamma interferon |
| β-NGF | beta nerve growth factor |
| SCF | stem cell factor |
| SCGF-β | stem cell growth factor-beta |
| SDF-1α | stromal cell-derived factor-1alpha |
| TNF-α | tumour necrosis factor-alpha |
| TNF-β | tumour necrosis factor-beta |
| TRAIL | TNF-related apoptosis-inducing ligand |
| FGF-basic | basic fibroblast growth factor |
| eotaxin | eosinophil chemotactic protein |
| G-CSF | granulocyte colony-stimulating factor |
| GM-CSF | granulocyte macrophage colony-stimulating factor |
| M-CSF | macrophage colony-stimulating factor |
| IP-10 | interferon gamma-induced protein-10 |
| MCP-1 | monocyte chemotactic protein-1 |
| MIP-1α | macrophage inflammatory protein-1 alpha |
| MIP-1β | macrophage inflammatory protein-1 beta |
| PDGF-BB | platelet-derived growth factor-BB |
| RANTES | regulated on activation, normal T cell expressed and secreted |
| VEGF | vascular endothelial growth factor |

**
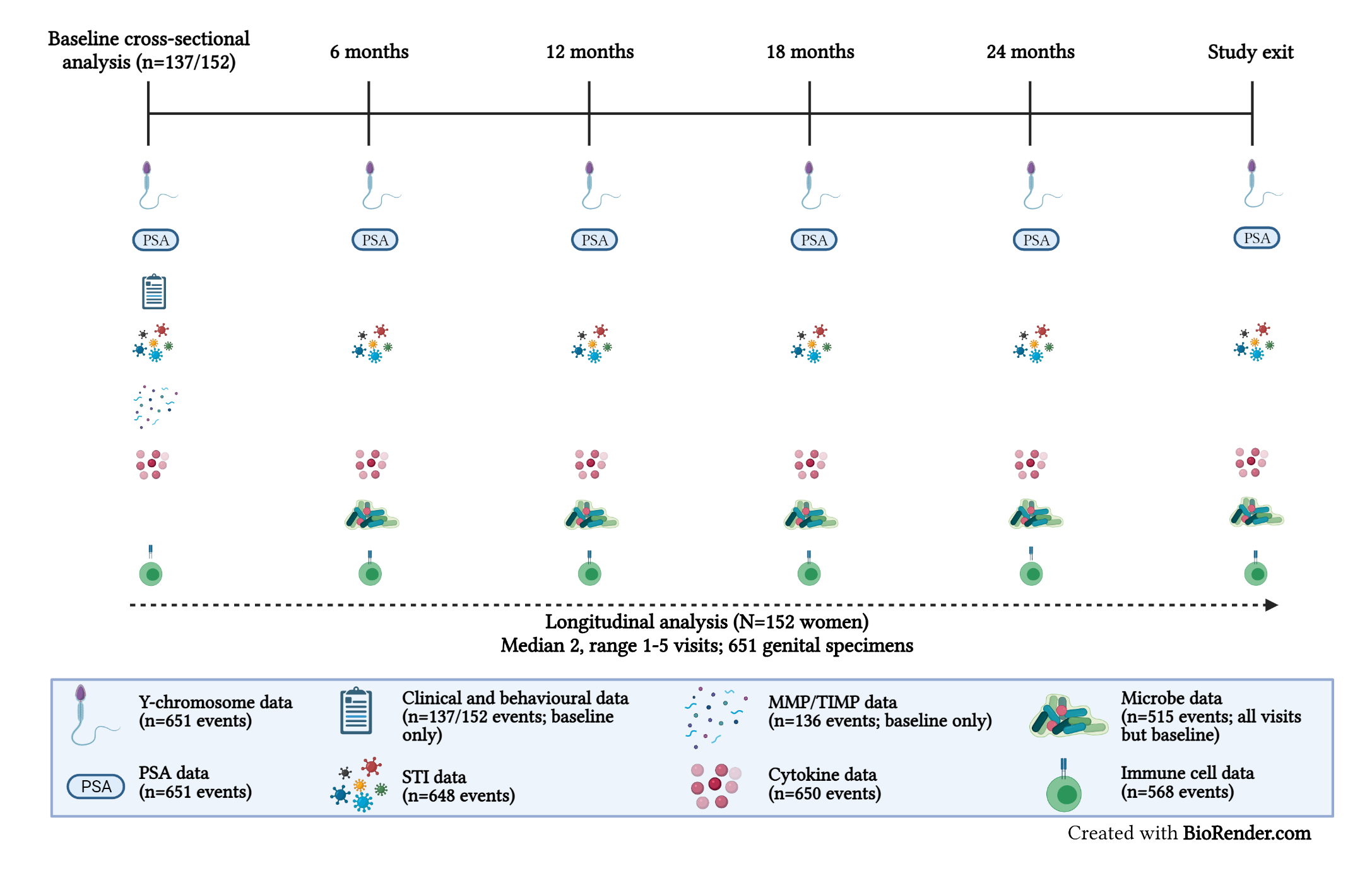
**

**Figure S1.** Graphical representation of the data available at baseline and longitudinally for CAPRISA 008 study participants. This study included 152 female participants from the CAPRISA 008 trial. Genital specimens were collected at baseline and longitudinally at months 6, 12, 18, 24, and study exit during the two-year trial (median 2, range 1-5 visits). The dataset included all baseline and longitudinal genital specimens with both YcDNA and PSA data available (n=651 genital specimens). Clinical and behavioural data are reported for the 137/152 women with both PSA and YcDNA data available at baseline. MMP/TIMP data were only available at baseline visits (n=136), and microbe data were available at all visits but baseline (n=515). PSA: Prostate-specific antigen; STI: sexually transmitted infection; MMP: matrix metalloproteinase; TIMP: tissue inhibitor of metalloproteinases

**Table S2.** Flow cytometry information on the antibody clones, fluorophores, and suppliers.

| **Antibody** | **Source** | **Identifier** |
| --- | --- | --- |
| Mouse anti-human CD14, Pacific Blue conjugated, clone M5E2 | BD | BD Biosciences  Cat# 558121,  RRID:AB_397041 |
| Mouse anti-human CD19, Pacific Blue conjugated, clone  HIB19 | BioLegend | BioLegend Cat#  302232,  RRID:AB_2073118 |
| Mouse anti-human CD8, Fluorescein isothiocyanate  (FITC) conjugated, clone SK1 | BioLegend | BioLegend Cat#  344704,  RRID:AB_1877178 |
| Mouse anti-human CD3, Allophycocyanin tandem dye  (APC-H7) conjugated, clone SK7 | BD | BD Biosciences  Cat# 560275,  RRID:AB_1645476 |
| Mouse anti-human CD4, Peridinin-chlorophyll protein-cyanine 5.5 (PerCP-Cy5.5) conjugated, clone RPA-T4 | BD | BD Biosciences  Cat# 560650,  RRID:AB_1727476 |
| Mouse anti-human CD38, eFluor655 conjugated, clone  HB7 | eBioscience (Thermofisher) | Thermo Fisher  Scientific Cat# 95-  0388-42,  RRID:AB_1724053 |
| Mouse anti-human HLA-DR, Phycoerythrin (PE)  conjugated, clone L243 | BD | BD Biosciences  Cat# 347401,  RRID:AB_2629277 |
| Mouse anti-human KI-67, Brilliant violet 700 (BV700)  conjugated, clone Ki-67 | BioLegend | BioLegend Cat#  350515,  RRID:AB_11218996 |
| Mouse anti-human CCR5, Allophycocyanin (APC)  conjugated, clone 2D7/CCR5 | BD | BD Biosciences  Cat# 556903,  RRID:AB_398619 |
| LIVE/DEAD Fixable Violet Dead Cell Stain (Intracellular  amines) | Invitrogen | Cat#34955 |

**Table S3.** List of common STI pathogens and other vaginal microbes measured in vulvovaginal swabs.

| **Abbreviation** | **STI pathogen/ vaginal microbe** |
| --- | --- |
| CT | *Chlamydia trachomatis* |
| TV | *Trichomonas vaginalis* |
| NG | *Neisseria gonorrhoeae* |
| MG | *Mycoplasma genitalium* |
| HSV-1 | Herpes simplex virus type 1 |
| HSV-2 | Herpes simplex virus type 2 |
| *L. crispatus* | *Lactobacillus crispatus* |
| *L. jensenii* | *Lactobacillus jensenii* |
| *G. vaginalis* | *Gardnerella vaginalis* |
| *P. bivia* | *Prevotella bivia* |
| BVAB2 | Bacterial Vaginosis-Associated Bacterium 2 |
| *Megasphaera 1* | *Megasphaera type 1* |
| *A. vaginae* | *Atopobium vaginae* |

**Table S4.** Sensitivity and specificity for the YcDNA concentration cutoff value of 0.005 ng/µl.

| **YcDNA concentration cutoff value** |  | **PSA** | | **Accuracy (95% CI)** |
| --- | --- | --- | --- | --- |
|  |  | Positive | Negative |  |
| **0.005 ng/µl** | Positive | 97 | 26 | Sensitivity = 87.4% (81.2 – 93.6)  Specificity = 62.3% (50.9 – 73.8)  PPV = 78.9% (71.7 – 86.1)  NPV = 75.4% (64.3- 86.6) |
|  | Negative | 14 | 43 |  |
| PPV: predictive value; NPV: negative predictive value; PSA: prostate-specific antigen; YcDNA: Y-chromosome DNA; CI: confidence interval | | | | |


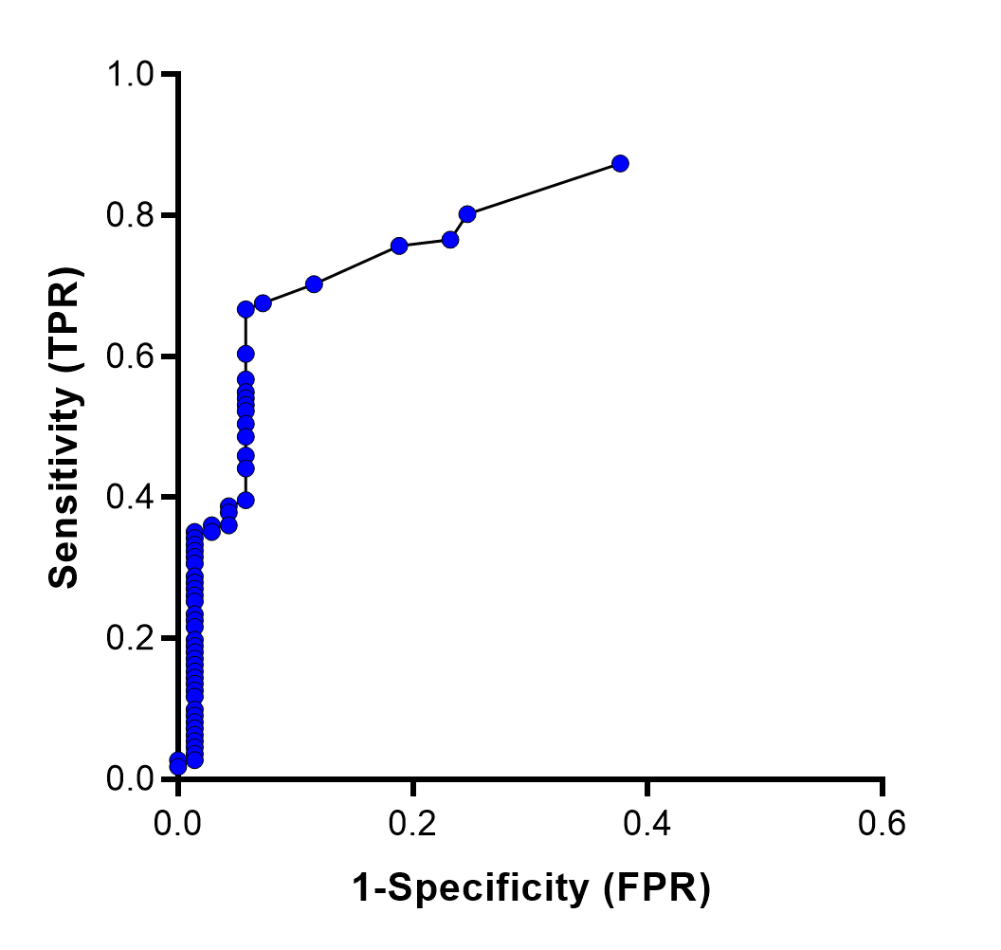


**Figure S2: ROC curve for all YcDNA concentration cutoff values.** Cutoff values ranged between 0.005 ng/µl and 1.700 ng/µl. TPR: true positive rate (sensitivity); FPR: false positive rate (1-specificity).
